# Nucleolin-binding by ErbB2 enhances tumorigenicity of ErbB2-positive breast cancer

**DOI:** 10.18632/oncotarget.11323

**Published:** 2016-08-17

**Authors:** Eya Wolfson, Maria Goldenberg, Shira Solomon, Amit Frishberg, Ronit Pinkas-Kramarski

**Affiliations:** ^1^ Department of Neurobiology, Tel-Aviv University, Ramat-Aviv, 69978, Israel; ^2^ Department of Cell Research and Immunology, Tel-Aviv University, Ramat-Aviv, 69978, Israel

**Keywords:** ErbB/HER family, nucleolin, tyrosine kinase, breast cancer, TCGA

## Abstract

ErbB2 is an important member of the ErbB family, which activates growth and proliferation signaling pathways. ErbB2 is often overexpressed in various malignancies, especially in breast cancer, and is a common target for anti-cancer drugs. Breast cancer is currently one of the leading mortality causes in women, and acquired resistance to ErbB2-targeted therapies is a major obstacle in its treatment. Thus, understanding ErbB2-mediated signaling is crucial for further development of anti-cancer therapeutics and disease treatment. Previously, we have reported that the ErbB receptors interact with the major nucleolar protein nucleolin. In addition to its function in the nucleoli of cells, nucleolin participates in various cellular processes at the cytoplasm and cell-surface. Deregulated nucleolin is frequently overexpressed on the membrane of cancer cells. Here, we show that nucleolin increases colony formation and anchorage-independent growth of ErbB2-overexpressing cells. Importantly, this enhanced tumorigenicity also occurs in human ErbB2-positive breast cancer patients; namely, nucleolin overexpression in these patients is associated with reduced patient survival rates and increased disease-risk. ErbB2-nucleolin complexes are formed endogenously in both normal and cancer cells, and their effect on tumorigenicity is mediated through activation of ErbB2 signaling. Accordingly, nucleolin inhibition reduces cell viability and ErbB2 activation in ErbB2-positive cancer cells.

## INTRODUCTION

The ErbB family of receptor tyrosine kinases (RTKs) mediates basic cellular processes, such as cell survival, proliferation and migration. This family includes four members: ErbB1 (EGFR), ErbB2 (HER2/neu), ErbB3 (HER3) and ErbB4 (HER4), which participate in signal transduction in response to extracellular stimuli [1]. Activation of these receptors occurs following ligand binding, which triggers receptor homo- or heterodimerization with other family members, leading to tyrosine phosphorylation of the cytoplasmic tail. As a result, various signaling pathways are activated, among which are the PI3K/Akt and Ras/MAPK pathways [2, 3]. Despite the fact that ErbB2 is an orphan receptor, it is one of the most active receptors of the family. Unlike other family members, it has an open conformation, with its dimerization loop constantly exposed, which facilitates the formation of an ErbB2 dimer with other adjacent ErbBs [2–4].

The ErbB receptors are often associated with malignant transformation [5]. High occurrence of hyper-active or overexpressed ErbBs is found in many cancer types, including skin melanoma, prostate, bladder, lung, bowel and breast cancers [2, 3, 6]. Since ErbB2 is more prone to dimerization than other family members, when amplified it is frequently involved in cancer development. Its overexpression is mostly common in breast cancer (~30% of patients), and was shown to play an important role in invasion of extra cellular matrix (ECM) by breast carcinoma [7].

Previously, we have found that endogenous ErbB family members bind to the nucleolar protein nucleolin [8]. Nucleolin is best known for its role in ribosomal biogenesis, though accumulating evidence indicate that it is also involved in many other processes, outside the nucleus, including cell differentiation and proliferation [9–11]. In addition, nucleolin exhibits a stress-conditioned activity through interactions with components of the stress-response machinery [10, 12–14]. It was also implicated in cellular shuttling, and in some cases was even found to act as a cell surface receptor [15, 16]. Nucleolin overexpression is linked to cancer progression. Its presence on the membrane of cancer cells is often elevated, and it was found to interact with tumor promoting proteins, such as VEGF and HGF [14]. Similarly, intracellular pools of nucleolin contribute to the tumorigenicity of cancer cells as well [17].

Having demonstrated that nucleolin binds ErbB receptors, in the present study, we have characterized the interaction between nucleolin and ErbB2 and its oncogenic effects. We have found that nucleolin promotes ErbB2 activation, and that overexpression of both proteins results in increased cell tumorigenicity. Moreover, overexpression of nucleolin is associated with enhanced disease progression and mortality rates in ErbB2-positive breast cancer patients. Accordingly, specific inhibition of nucleolin by shRNA transfection, competitive peptide binding or GroA drug treatment [18] impeded the observed cellular phenomena. Thus, we propose a novel pathway for ErbB2-activation, which promotes oncogenic transformation, and affects tumor growth and disease progression. Targeting this interaction could provide a basis for the development of new anti-cancer drugs, which may help overcome acquired tumor-resistance to ErbB2-targeted drugs [19, 20].

## RESULTS

### ErbB2 and nucleolin physically interact in intact cells

**Figure 1:**
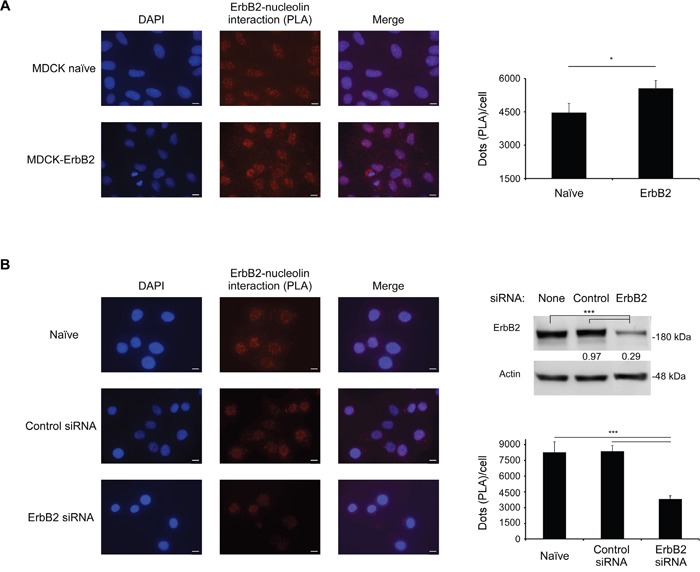
ErbB2 and nucleolin interact in intact cells **A.** *Left panel*, visualization of the interaction between ErbB2 and nucleolin (red dots) in naïve MDCK cells and MDCK ErbB2-expressing clones was performed using a proximity ligation assay (PLA). *Right panel*, differences between signal intensity in both cell lines represented as the number of dots per cell (means ±SE). **B.** *Left panel*, visualization of the interaction between ErbB2 and nucleolin (red dots) in SKBR3 untreated cells and cells treated with either control siRNA or ErbB2-specific siRNA. *Upper right panel*, a western-blot analysis of ErbB2-siRNA treatment effect on ErbB2 levels in SKBR3 cells; numbers below bands indicate average fold induction of untreated. *Lower right panel*, differences in signal intensity represented as number of dots per cell (means ±SE).

Previously, using co-immunoprecipitation (co-IP) analysis, we have shown that endogenous nucleolin and ErbB2 interact [8]. In order to confirm this interaction and to determine its endogenous localization in intact cells, we performed a proximity ligation assay (PLA) [21]. Results obtained from naïve and ErbB2-overexpressing non-cancerous MDCK cells have, indeed, confirmed this interaction, which was detected throughout the cell: near the cell periphery, in the cytoplasm and in the nucleus (Figure 1A). As expected, the intensity of the signal measured was significantly higher for the ErbB2-overexpressing cells than for the naïve cells. A similar pattern was observed in SKBR3 breast cancer cells, which endogenously express high levels of ErbB2: here, introduction of siRNA targeted against ErbB2 has significantly reduced ErbB2-nucleolin interaction, compared to untreated cells or cells treated with a non-specific control siRNA (Figure 1B). Therefore, we have concluded that, indeed, ErbB2 directly binds nucleolin, and that this interaction occurs endogenously in normal and cancer cells.

### Overexpression of nucleolin and ErbB2 increases colony formation and anchorage-independent growth of MDCK cells

**Figure 2:**
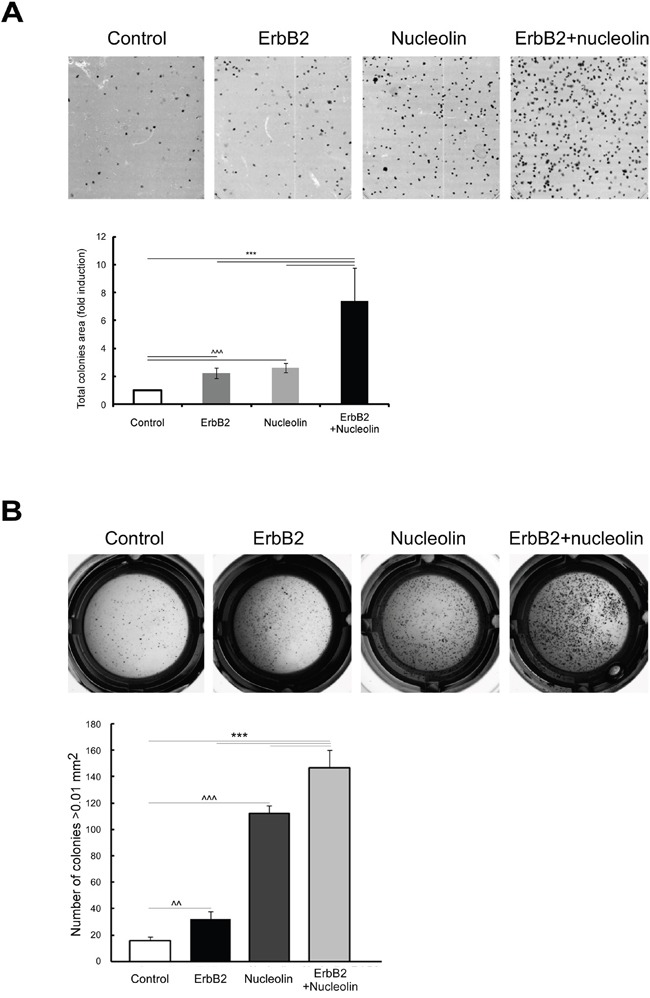
Overexpression of both nucleolin and ErbB2 increases cell tumorigenicity **A.** Colony formation was tested in either naïve MDCK cells or MDCK stable clones expressing ErbB2 and nucleolin, either separately or in combination. *Upper panel*, representative images. *Lower panel*, total colonies area presented as fold induction of control (means ±SD). **B.** MDCK cells overexpressing nucleolin, ErbB2, nucleolin and ErbB2 or none were plated and grown in soft agar, as described in Materials and Methods. *Upper panel*, photomicrographs of typical wells. *Lower panel*, number of colonies (size>0.01 mm^2^; means ±SD). *** (*p-value*<0.005) - ErbB2 and nucleolin clone compared to the controls; ^^^ (*p-value*<0.005) - ErbB2/nucleolin clones compared to naïve control.

Since the association between ErbB2 and nucleolin appears to naturally occur in breast cancer cells, and since we have previously shown that the interaction between nucleolin and ErbB1 enhances cell growth [8], we examined the effects of combined nucleolin and ErbB2 overexpression on cell growth and tumorigenicity. In order to test the effect of the two proteins on colonies formation, we have performed a long-term clonogenic assay. For this purpose, we have used MDCK cells stably expressing high levels of either ErbB2, nucleolin or both (naïve MDCK cells were used as a control). Overexpression of either ErbB2 or nucleolin alone resulted in a significant increase in the total colonies area compared to the control cells. This effect was further enhanced by the overexpression of both proteins together (Figure 2A).

Next, we examined whether the overexpression of each protein alone or both together affects anchorage independent growth. Cells were plated and grown in soft agar, and the number of colonies formed was quantified. Cells overexpressing either ErbB2 or nucleolin alone formed a significantly greater number of colonies, compared to the naïve control cells. An additional, significant, increase in colonies number was observed in cells overexpressing both proteins (Figure 2B). Taken together the results indicate that co-expression of nucleolin and ErbB2 may contribute to oncogenic transformation.

### Identification of the nucleolin ErbB2-interacting region

**Figure 3:**
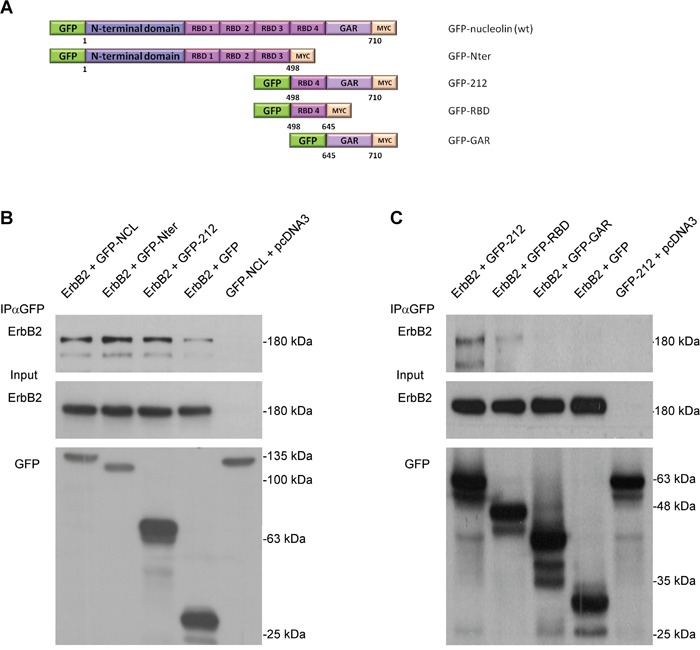
Nucleolin associates with ErbB2 through its RNA-binding domains **A.** Schematic representation of full-length (wt) nucleolin and its truncation variants: N-ter, 212, RBD and GAR. **B.** Co-immunoprecipitation (co-IP) analysis of ErbB2 and full-length nucleolin (NCL), N-ter and 212. HEK-293T cells were co-transfected with ErbB2 and either nucleolin variants or control vectors. **C.** Co-IP analysis of ErbB2 and the C-terminal variants of nucleolin: 212, RBD and GAR. HEK-293T cells were co-transfected with ErbB2 and either 212 (as a positive control), RBD, GAR or control vectors.

To further explore the nature of nucleolin-ErbB2 interaction, we determined the ErbB2-binding region of nucleolin by performing a co-IP assay using nucleolin truncation variants (Figure 3A). HEK-293T cells were co-transfected with vectors encoding for ErbB2 and either GFP-tagged wt nucleolin, N-ter or C-ter-212 (212) truncation proteins. Intriguingly, though ErbB1 appeared to interact exclusively with the 212 variant [22], ErbB2 was precipitated by both nucleolin variants (Figure 3B). This could be explained by the presence of RNA Binding Domains (RBDs) in both truncated nucleolin proteins, and is further supported by the fact that the RBD region located within the 212 C-terminal amino acids of nucleolin contributes to ErbB1-binding [22].

To test this, we have used two additional nucleolin variants, each representing a different portion of the 212 variant; a variant containing the fourth RBD region and a variant containing only the GAR domain, lacking any RBD sequences (RBD, GAR, respectively; Figure 3A). Indeed, ErbB2 appeared to co-precipitate only with the RBD variant (Figure 3C).

### Identification of the ErbB2 nucleolin-interacting region

**Figure 4:**
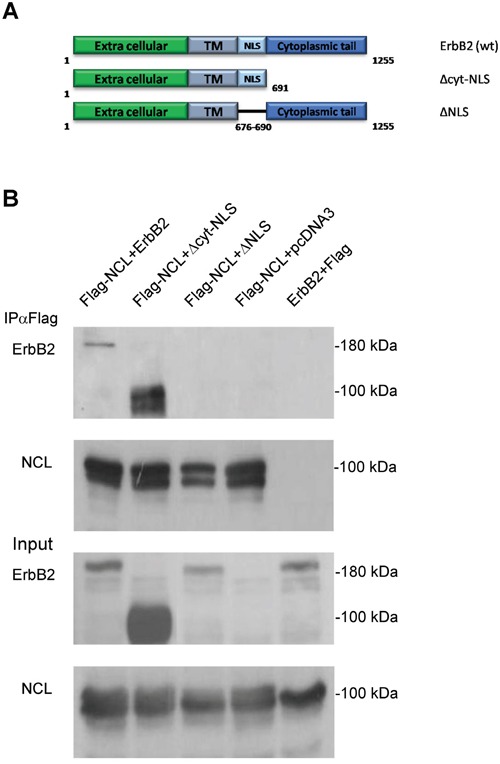
The NLS domain of ErbB2 is responsible for nucleolin-binding **A.** Schematic representation of full-length (wt) ErbB2 and its mutants: Δcyt-NLS and ΔNLS. **B.** Co-IP analysis of nucleolin and ErbB2 mutants. HEK-293T cells were co-transfected with Flag-tagged nucleolin and either wt ErbB2, ΔNLS, Δcyt-NLS or control vectors.

Nucleolin is known to bind the NLS domain of many of its interacting proteins [12, 23], including ErbB1 [22]. Therefore, in order to identify the ErbB2 domain responsible for nucleolin-binding, we have generated two deletion mutants of the receptor: ΔNLS and Δcyt-NLS (Figure 4A), lacking either the NLS region or the cytoplasmic tail, respectively. HEK-293T cells were co-transfected with Flag-tagged nucleolin and either wt ErbB2, ΔNLS or Δcyt-NLS, and subjected to co-IP analysis. The results indicated that while nucleolin was able to bind both full-length ErbB2 and Δcyt-NLS, it failed to precipitate the ΔNLS mutant, suggesting that the NLS is essential for the interaction (Figure 4B).

### The N-terminal and 212 C-terminal domains of nucleolin increase ErbB2-phosphorylation

**Figure 5:**
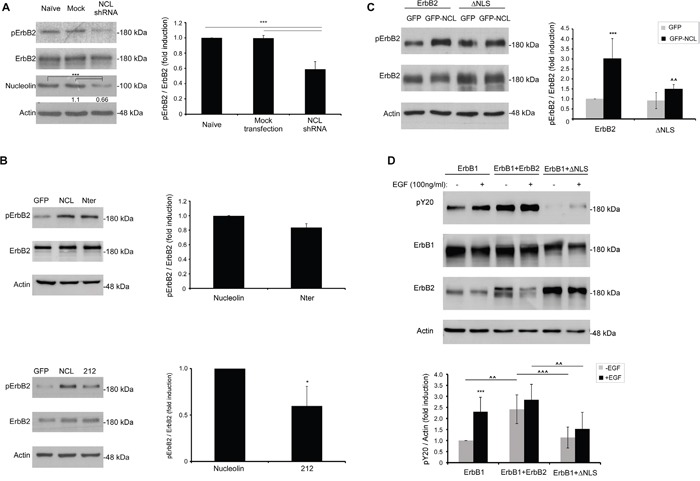
Nucleolin and its truncation variants enhance ErbB2 ligand-independent phosphorylation Phosphorylation levels of ErbB2 were determined by western-blotting using an anti-phospho-ErbB2 antibody. **A.** SKBR3 cancer cells were transiently transfected with anti-nucleolin shRNA; numbers below bands indicate average fold induction of nucleolin relative to untreated cells (means ±SD). **B.** HEK-293T cells were transiently transfected with ErbB2 and either wt nucleolin, N-ter, 212 or a control vector (means ±SD). **C.** HEK-293T cells were transiently transfected with ErbB2 or ΔNLS and either wt nucleolin or a control vector (means ±SD; *** (*p-value*<0.005) - nucleolin-transfected compared to GFP-transfected control; ^^ (*p-value*<0.01) -ΔNLS-transfected compared to wt ErbB2-transfected cells). **D.** HEK-293T cells were transiently transfected with either ErbB1 alone or in combination with wt ErbB2 or ΔNLS, and total phosphorylation levels of Y20 were measured by western-blotting, prior to and following EGF-stimulation (10min), as indicated (means ±SD; *** (*p-value*<0.005) - EGF-stimulated compared to unstimulated cells; ^^^ (*p-value*<0.005) - comparison between co-transfections in cells either unstimulated or stimulated with EGF).

We have demonstrated previously that ectopic overexpression of full-length nucleolin triggers ligand-independent phosphorylation of ErbB2 [8]. We, therefore, examined the effect of endogenously expressed nucleolin on ErbB2 activation in SKBR3 cells. Cells were either mock transfected or transfected with anti-nucleolin shRNA, and ErbB2 phosphorylation levels were determined by western-blot analysis. Indeed, we have found that nucleolin depletion led to a significant decrease in phosphorylated ErbB2 (Figure 5A). Next, since both the N-ter and the 212 variants were found to bind ErbB2, they were used to further identify the region of nucleolin responsible for receptor phosphorylation. The effect on ErbB2 phosphorylation was assessed by western-blot analysis of HEK-293T cells co-transfected with ErbB2 and either nucleolin, 212, N-ter or a control vector (Figure 5B). Though both nucleolin variants were able to phosphorylate ErbB2, while the N-ter variant led to only slightly reduced phosphorylation, compared to wt nucleolin, the effect of the 212 variant was significantly lower.

### The NLS domain of ErbB2 is required for nucleolin-dependent activation and receptor kinase activity

Additional evidence for the importance of the NLS domain of ErbB2 for nucleolin-binding was obtained by measuring activation of the ΔNLS mutant in the presence of wt nucleolin. Phosphorylation levels of ErbB2 were measured in HEK-293T cells co-expressing wt ErbB2 or ΔNLS and either nucleolin or GFP control-vector. Phosphorylation of the ΔNLS mutant in the presence of nucleolin was significantly lower than that of wt ErbB2 (Figure 5C). Hence, physical binding of nucleolin is essential for ErbB2-activation.

Previously, we have shown that in ErbB1, the NLS domain is required for nucleolin-independent EGF-induced receptor activation [22, 24]. We, therefore, tested whether the NLS of ErbB2 has a similar function, by overexpressing ErbB1 alone or in combination with either ErbB2 or ΔNLS in EGF-stimulated cells. Indeed, while ErbB2 seemed to increase the overall phosphorylation rates of tyrosine residues in the cells, phosphorylation levels were significantly lower in cells expressing the ΔNLS mutant, even following EGF stimulation (Figure 5D). Since EGF-induced receptor phosphorylation depends on the activity of the two kinases within the heterodimer, this suggests that the NLS domain of ErbB2 is required for proper ErbB2 kinase function.

### ErbB2 binding by nucleolin initiates signal transduction through the MAPK pathway

**Figure 6:**
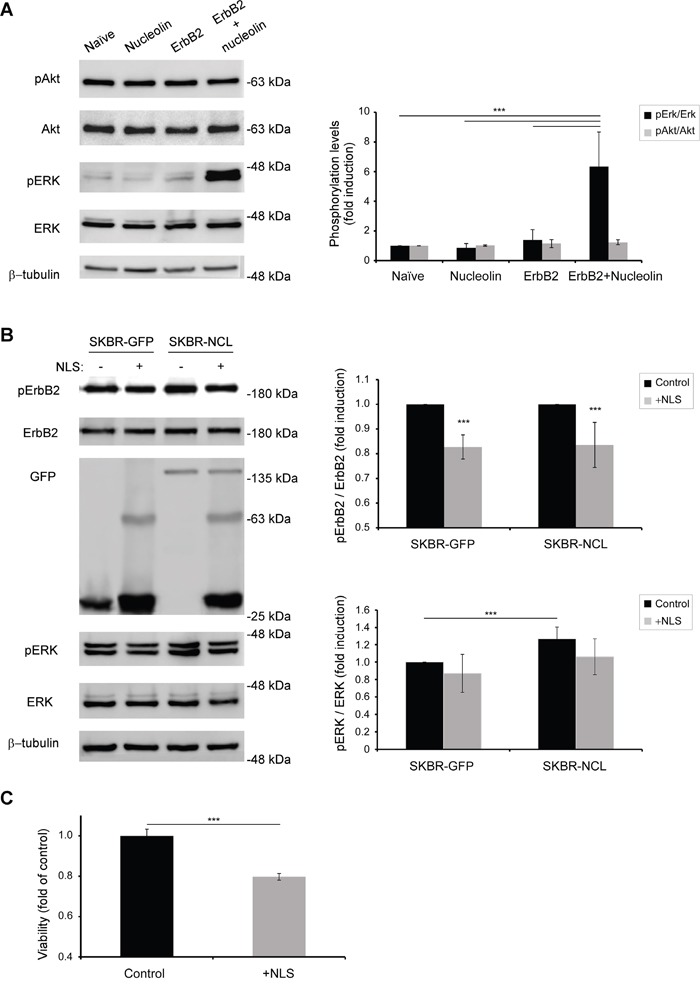
Binding of nucleolin specifically activates ErbB2 and the MAPK pathway **A.** Western-blot analysis of activation levels of ErbB2 downstream signaling pathways, as determined by phosphorylation of Erk and Akt proteins in HEK-293T cells transiently transfected with ErbB2, nucleolin, neither or both (means ±SD). **B.** Activation of ErbB2 by nucleolin and its downstream signaling was inhibited by transient overexpression of GFP-TM-NLS peptides in stably GFP or GFP-nucleolin-overexpressing SKBR3 breast cancer cells (means ±SD). **C.** SKBR3 cells were transiently transfected with either GFP-TM-NLS or a control, and cell viability was measured using the methylene blue assay (means ±SD).

Since ErbB2 signaling affects two major signaling pathways: PI3K and MAPK [25], we have tested whether excess of nucleolin and ErbB2 causes activation of either pathway. Western-blot analysis performed on HEK-293T cells, transiently transfected with either ErbB2, nucleolin or both, has shown an increase in phosphorylation levels of Erk in the presence of both proteins, compared to each protein alone; the phosphorylation levels of Akt, however, remained unchanged (Figure 6A). These results suggest that nucleolin facilitates ErbB2-mediated activation of the MAPK pathway, whereas, in this cell line, the PI3K pathway is largely unaffected by both proteins. Similar increase in MAPK activation in the presence of overexpressed nucleolin was observed in SKBR3 cells (Figure 6B).

We then explored the effect of ErbB2-nucleolin interaction inhibition on ErbB2 activation and signaling. As described by us previously [22] and in Figures 4 and 5, the NLS domains of ErbB1 and ErbB2 are responsible for nucleolin binding and consequent receptor activation. Given that both receptors are highly homologous [3, 22], we have used a GFP-tagged, truncated, variant of ErbB1, consisting only of its signal peptide sequence, transmembrane domain and NLS (termed GFP-TM-NLS) as a competitor for nucleolin binding. Theoretically, excess of such GFP-TM-NLS peptides would result in an impairment of the ErbB2-nucleolin association and its downstream signaling, even in the presence of high nucleolin levels. SKBR3 clones stably expressing either GFP (SKBR-GFP) or GFP-nucleolin (SKBR-NCL) were transiently transfected with the GFP-TM-NLS vector. Following the expression of GFP-TM-NLS, phosphorylation levels of ErbB2 appeared to decrease in both clones, indicating that the interaction was inhibited by the ErbB1-NLS peptide, even in cells overexpressing nucleolin (Figure 6B). Importantly, GFP-TM-NLS expression resulted in a significant decrease in SKBR3 cells viability, indicating that the observed signaling inhibition was sufficient to suppress cell growth (Figure 6C).

### Nucleolin affects disease risk in ErbB2-positive breast cancer patients

**Figure 7:**
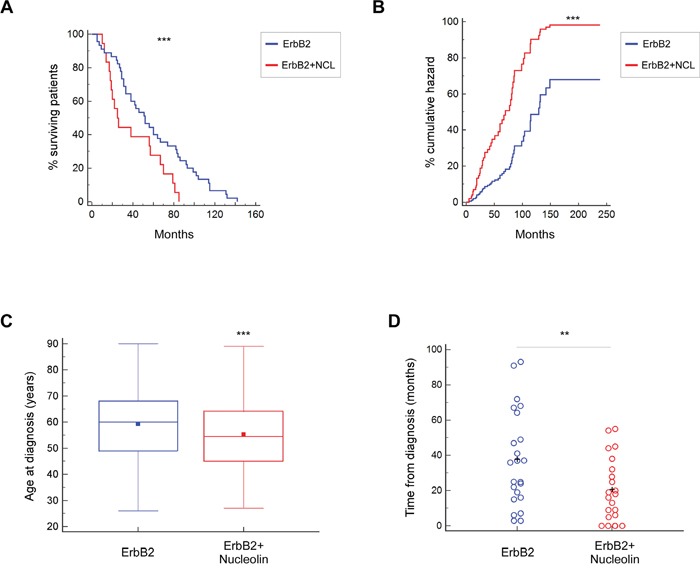
Overexpression of nucleolin in ErbB2-positive breast cancer patients is associated with earlier onset and poor prognosis **A.** Kaplan-Meier curves depicting survival rates of patients overexpressing either ErbB2 alone or both ErbB2 and nucleolin (n=64). **B.** Cox-proportional hazards model for ErbB2-positive breast cancer patients, with or without nucleolin overexpression (n=305). **C.** Age of disease onset in patients overexpressing either ErbB2 alone or both ErbB2 and nucleolin (n=518). **D.** Time from initial diagnosis until detection of an additional, new, tumor (metastatic or primary) in the same patient (n=42). Data was compared between patients overexpressing ErbB2 and patients overexpressing ErbB2 and nucleolin.

After determining that ErbB2 signaling is enhanced by nucleolin, which increases cell growth, we decided to test for a similar impact in humans. Breast cancer patients often express high levels of ErbB2. Such expression is correlated with poor prognosis and multi-drug resistance [20, 26]. Though the role of this receptor in breast cancer is vastly explored, the additional impact of nucleolin overexpression on tumor progression in human patients remains unknown. Using clinical and transcriptional data from breast cancer patients obtained from the TCGA Research Network, we have performed bioinformatical analyses comparing disease onset and progression in either ErbB2-positive or ErbB2 and nucleolin-positive breast cancer patients. Our analysis indicated that the overall survival time was significantly shorter for patients with both ErbB2 and nucleolin overexpression (Figure 7A), and that this genotype was also associated with higher risk (Figure 7B). Moreover, it appears that in patients overexpressing both proteins, the age of disease onset is significantly younger compared to patients overexpressing ErbB2 alone (55.27 and 59.23 years, respectively; Figure 7C). The difference between these two groups was further emphasized by the fact that the average time from initial diagnosis to development of a new tumor was significantly shorter in the double-positive patients (Figure 7D). Thus, the data suggest that nucleolin overexpression correlates with poor prognosis and increased risk of breast cancer in ErbB2-positive patients.

### Nucleolin inhibition by GroA affects viability and growth of SKBR3 breast cancer cells

**Figure 8:**
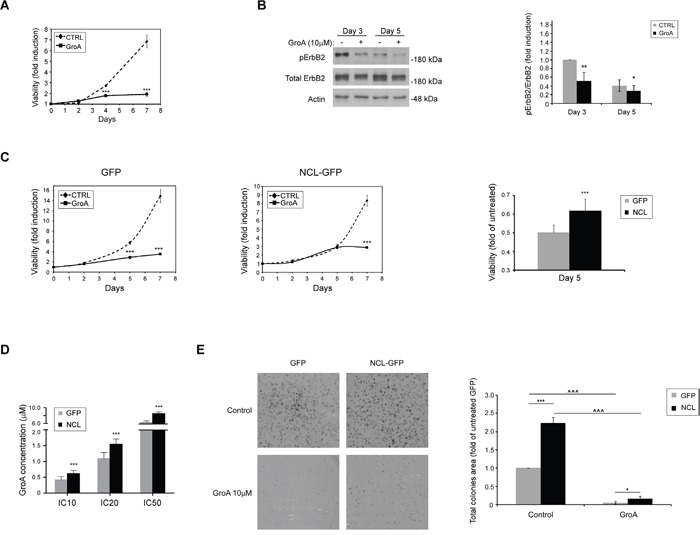
The nucleolin-targeted drug GroA (AS1411) inhibits cell growth and ErbB2 activation **A.** SKBR3 cells were treated with GroA (10μM), and cell viability was measured by the methylene blue assay at the indicated time points (means ±SD). **B.** Western-blot analysis of ErbB2 activation, determined by ErbB2 phosphorylation levels, following treatment with GroA (means ±SD). **C.** SKBR3 cells overexpressing either GFP-nucleolin (SKBR-NCL) or GFP (SKBR-GFP) were treated with GroA (10μM), and cell viability was measured using the methylene blue assay. *Left panel*, time course curves of cell viability following treatment in SKBR-GFP (control) and SKBR-NCL cells. *Right panel*, comparison of cell viability in SKBR-GFP and SKBR-NCL cells after 5 days of treatment. Results presented as means ±SD. **D.** The IC-10, 20 and 50 values for GroA treatment were determined for SKBR-NCL and SKBR-GFP cells using the methylene blue viability assay (means ±SD). **E.** Cells were pre-treated with GroA as indicated, and total area of colonies formed was determined (means ±SD; *** (*p-value*<0.005) - SKBR-GFP compared to SKBR-NCL cells; ^^^ (*p-value*<0.005) - comparison between untreated and treated cells of the same cell line).

As it appears that high nucleolin levels are associated with cancer development in ErbB2-positive breast cancer patients, and since anti-nucleolin RNAi and competitive inhibition of nucleolin by binding of GFP-TM-NLS peptides appeared to reduce ErbB2 activation, we hypothesized that treatment with nucleolin-targeting drugs could decrease breast cancer cell growth. In order to test this hypothesis, SKBR3 cells were treated for 2, 4 or 7 days with GroA (AS1411), a G-rich aptamer, which selectively inhibits nucleolin. Previously, GroA was shown to have an inhibitory effect on colorectal cancer and glioblastoma cells [27, 28], and was used in phase II clinical trials of acute myeloid leukemia (AML) [18, 29, 30]. Cell viability was measured at the indicated time points. The growth rates of treated cells seemed to drop drastically, as early as 4 days post-treatment, compared to the control cells (Figure 8A). Moreover, treated cells exhibited a decrease in active receptor levels, compared to untreated cells (Figure 8B).

In order to further examine the role of nucleolin in SKBR3 cells, we have compared the effects of GroA treatment on SKBR-GFP and SKBR-NCL cells. SKBR-NCL cells were less responsive to this treatment than control (SKBR-GFP) cells (Figure 8C and 8D). While the control cells were markedly affected by the treatment after 3 days, a similar decrease in viability of GroA-treated nucleolin-overexpressing cells was only visible after more than 5 days. This was further supported by increased inhibitory concentrations (IC)-10, 20 and 50 values in the SKBR-NCL cells. Though these cells were still affected by the treatment, they seem to have acquired a certain resistance, compared to the control SKBR-GFP cells. Similar results were obtained from colony formation experiments. Cells treated with GroA formed fewer and much smaller colonies than untreated cells, as evident by total colony area; here, as well, the SKBR-NCL cells appeared less susceptible to the treatment than the control cells (Figure 8E). This might be explained by the excess of nucleolin introduced into the cell, which could enhance the occurrence of the ErbB2-nucleolin interaction, by shifting the balance between GroA-bound and free nucleolin. Nonetheless, these results suggest that nucleolin can be used as a novel target in development of anti-cancer treatments for ErbB2-positive breast tumors.

## DISCUSSION

As a member of the RTK ErbB family, ErbB2 is a major contributor to cell survival, proliferation and migration signaling. Phosphorylated ErbB2 transmits the signal to downstream pathways, such as PI3K and MAPK [2, 3]. Abnormal expression of this receptor is linked with malignant transformation, with breast cancer being the most common type of ErbB2-driven malignancy [2, 20]. Similarly, nucleolin, a protein best known for its nucleolar functions, is also highly implicated in cancer onset and development [14]. Nucleolin is a multifunctional protein, and apart from its involvement in transcription of rRNA and ribosomal assembly, it was also reported to participate in cellular functions outside the nucleus [10, 11]. We have previously demonstrated that endogenous nucleolin binds all four ErbB receptors, and that ectopic expression of nucleolin triggers ligand-independent activation of both ErbB1 and ErbB2 [8]. Here, we have characterized the interaction between nucleolin and the most oncogenic family member, ErbB2.

The effects of nucleolin and ErbB2 co-expression may be the result of the physical interaction between these two proteins. Such interaction appears to occur endogenously, in cancer and normal cells, and is localized to several intracellular sites: near the cell surface, in the cytosol and in the nucleus; the latter being consistent with the known cellular distribution of nucleolin [14] and a previously reported nuclear localization of ErbB2 [31, 32]. We have found that the NLS region of ErbB2 binds nucleolin through one, or more, of its RBDs. This is in accordance with our previous finding of a similar association between nucleolin and ErbB1 [22], as well as with another report showing that the RBD of nucleolin binds the NLS sequence of Hdm2 [12, 23, 33]. As both the N-terminal region of nucleolin (N-ter variant), containing three out of its four RBDs, and the C-terminus (212 variant), containing the fourth RBD, were found to associate with the receptor, it remains unclear which of the RBDs are responsible for binding of full-length nucleolin; however, we did find that a single RBD is capable of and is sufficient for the binding.

We have discovered that formation of nucleolin-ErbB2 complexes results in phosphorylation of ErbB2, and the downstream activation of the MAPK pathway, but not the PI3K pathway. Importantly, depletion of endogenous nucleolin in ErbB2-positive SKBR3 breast cancer cells resulted in a significant impairment of ErbB2 phosphorylation. In addition, nucleolin was not able to induce phosphorylation of the ErbB2 NLS-deletion mutant (ΔNLS), suggesting that the observed activation is a result of the physical interaction between the proteins. Accordingly, ErbB2-activation could also be induced by each of the nucleolin truncation variants; however, while the presence of the N-ter variant had a similar effect compared with wt nucleolin, the effect of the 212 truncation variant was rather impaired. This could be due to the difference in the number of RBDs between the two nucleolin variants. As we reported previously, nucleolin is able to stabilize and trigger a ligand-independent phosphorylation of ErbB1 as well; however, analysis of the receptor activation in the presence of the N-ter and 212 variants revealed that only the latter was able to bind and activate ErbB1 [22]. A possible explanation for such discrepancy between the receptors could lie in their different conformations: while, when no ligand is present, ErbB1 is found predominantly in a closed conformation, ErbB2 possesses a constantly open conformation [4], which could account for differential nucleolin-binding.

We have previously observed that EGF-stimulation of ErbB1 receptors lacking an NLS sequence resulted in receptor dimerization, but not in its consequent phosphorylation [22]; since ErbB1 and ErbB2 possess highly similar structures [3], the lack of phosphorylation of the ErbB2-ΔNLS mutant in the presence of nucleolin could stem from some additional reasons, besides ablation of its nucleolin-binding abilities. Indeed, we have found that the overall phosphorylation levels in cells exhibiting ErbB1 alone or in combination with ErbB2-ΔNLS (prior to- and following EGF-stimulation) were significantly lower than in cells expressing ErbB1 and wt ErbB2, indicating that the NLS region affected receptor activation not only through nucleolin-binding. Hence, we propose that deletion of the NLS of ErbB2 leads to impaired kinase function of the receptor; since the NLS is required for proper kinase function in ErbB2, and is also the nucleolin-binding region, it is possible that binding of nucleolin to the NLS sequence activates the receptor's kinase, which allows tyrosine-phosphorylation of the receptor.

We found that expression of an ErbB1 deletion-mutant, which lacks both the extra- and intracellular domains, and consists only of the signal-peptide sequence, transmembrane domain and the NLS region (GFP-TM-NLS), leads to a decrease in ErbB2 phosphorylation levels in SKBR3 cells. This decrease was evident both in the SKBR-GFP control cells and in the SKBR-NCL nucleolin-overexpressing cells, and was similar between both cell lines. Though the GFP-TM-NLS peptide still retains the nucleolin-binding ability of ErbB1, it has no ability to form a dimer with the endogenous ErbB2. Hence, its interference with ErbB2 phosphorylation may be a result of its interaction with nucleolin, which depletes the amount of free nucleolin proteins available for ErbB2-binding, further emphasizing the effect of nucleolin on ErbB2 activation. Furthermore, GFP-TM-NLS expression sufficed to significantly impair SKBR3 cell viability.

Collectively, our results indicate that the ErbB2-nucleolin interaction occurs naturally in mammalian cells, and has an oncogenic potential. This phenomenon might not be limited to cell cultures, since bioinformatical analysis of over 500 patient samples obtained from the TCGA Research Network had revealed that nucleolin overexpression appears to have a significant additional impact on disease progression in ErbB2-positive tumors. Death and risk rates of patients whose tumors exhibited high levels of both proteins were found to be significantly higher than those of patients who had only overexpressed ErbB2. This enhanced effect is not limited to the outcome of the disease, but also affects its development, as disease onset appeared to occur earlier in ErbB2-nucleolin double-positive patients. Moreover, in these patients, the time required for the occurrence of a new tumor since the initial diagnosis was significantly shorter. Thus, seemingly, high levels of nucleolin and ErbB2 have a combined effect not only *in vitro*, but also *in vivo*, in humans.

Importantly, treatment of SKBR3 cells with the nucleolin-specific inhibitor GroA (AS1411) has significantly inhibited cell viability and colony formation, and led to a decrease in ErbB2 activation. These effects were somewhat mitigated in SKBR-NCL cells, where nucleolin is more abundant, and a higher dosage of GroA is required to obtain inhibition rates similar to those observed in control SKBR3 cells. This is in accordance with the results obtained following RNAi against nucleolin and administration of GFP-TM-NLS to the cells, further indicating the relation between nucleolin-binding and activation of ErbB2 and its downstream signaling pathways. Notably, GroA was previously used in phase I and II clinical trials of acute myeloid leukemia (AML), and was found promising. Thus, our results suggest GroA should be further examined as a potential new treatment for ErbB2-positive breast cancer.

Taken together, our findings indicate that nucleolin is an active participant in ErbB2 activation and initiation of ErbB2-mediated signal-transduction. Overexpression of ErbB2 and nucleolin leads to increased activation of the former and its downstream signaling through physical interaction between these two proteins. This might result in increased cellular growth and tumorigenicity, which develop into malignant transformation and lead to tumor formation; according to our analysis of breast cancer patients' data, occurrence of such double overexpression correlates with higher risk and mortality rates. It is yet unclear, what are the exact physiological roles of nucleolin-ErbB2 complex formation in normal cells, and through which cellular mechanisms, other than the MAPK signaling pathway, these functions are carried out. In this regard, ErbB2 is known to enhance neuregulin signaling through heterodimerization with ErbB3; such heterodimers play a central role in the transformation and development of ErbB2-positive tumors [34]. Moreover, overexpression of both proteins in cancer patients was found to correlate with poor prognosis [6]. The interplay between ligand-mediated and nucleolin-mediated ErbB signaling remains to be elucidated, and further investigations as to whether nucleolin is involved in ErbB2-ErbB3 dimer signaling, or is a part of an independent ErbB2-signaling mechanism, are needed.

According to the GLOBOCAN 2012 report, breast cancer is the most common malignancy in women, with the second highest mortality rates after lung cancer [35]. The high incidence of ErbB2 abnormal expression in this disease makes the involvement of nucleolin in ErbB2 signaling especially intriguing, and offers new insights on the development and progression of ErbB2-driven breast cancer, as well as on novel targets for cancer therapy. Moreover, since ErbB2 is also known to play a major role in other cancers, such as ovarian cancer [36, 37], it is possible that nucleolin might be involved in ErbB2 activation in this disease as well, and might affect the progression of this malignancy. Thus, further research should be performed in order to fully understand the impact of nucleolin-mediated ErbB2 signaling, and its possible implications on cancer therapy of ErbB2-positive cancers.

## MATERIALS AND METHODS

### Materials and buffers

The antibodies used are as follows: monoclonal mouse anti-actin (691001; MP Biomedicals, Santa Ana, CA); polyclonal rabbit anti-ErbB2 (HER2/neu), rabbit anti-nucleolin (C23), rabbit anti-Erk2 and rabbit anti-Akt, monoclonal mouse anti-phosphotyrosine (pY20) and monoclonal mouse anti-GFP and mouse anti-nucleolin (sc-284; sc-13057; sc-154; sc-8312; sc-508; sc-9996; sc-8031, respectively; Santa Cruz Biotechnology, Dallas, TX); monoclonal mouse anti-tubulin (T7816; Sigma-Aldrich); polyclonal rabbit anti-phospho-ErbB2, rabbit anti-phospho-Akt, rabbit anti-phospho-Erk1/2 (2249; 4058; 9101, respectively; Cell Signaling Technology, Danvers, MA); and monoclonal mouse anti-ErbB2 (extracellular; L87), which was a gift from Prof. Y. Yarden, Weizmann Institute of Science, Israel.

The aptamer GroA (AS1411) and the inactive oligomer Cro, were purchased from IDT (Jerusalem, Israel) as unmodified desalted oligonucleotides, as previously described [38].

### Cell lines

MDCK, HEK-293T cells and human breast cancer cell line SKBR3 were all grown in Dulbecco's modified Eagle's medium (DMEM; Biological Industries, Beithaemek, Israel). All media were supplemented with antibiotics and 10% heat-inactivated fetal bovine serum (FBS; Hyclone, Thermo Scientific). Cells were incubated at 37°C in 5% CO_2_ in air, and the medium was changed every 3–4 days. When 70% confluent, cells were passaged in trypsin/disodium ethylenediaminetetraacetic acid (Biological Industries, Beithaemek, Israel). One day before treatment the cells were plated at ~50% confluence in medium supplemented with 10% FBS.

### DNA and siRNA transfections

MDCK and SKBR3 cells were stably transfected with the jetPEI reagent (Polyplus transfection, New York, NY), according to the manufacturer's instructions. Stable clones expressing GFP, nucleolin, ErbB2 or both ErbB2 and nucleolin were selected and cultured with 1 mg/ml geneticin (GFP, nucleolin; G-418, Calbiochem, San Diego, CA) or/and 1 μg/ml puromycin (ErbB2; Sigma-Aldrich). At least three clones from each transfection were chosen for further use. Transient transfections were performed either by using jetPEI reagent (HEK-293T, SKBR3) or through calcium phosphate (CaPO_4_; HEK-293T) precipitation. Transiently transfected cells were harvested for further analysis 48-72h post-transfection.

Anti-nucleolin shRNA (62-320; Upstate), anti-ErbB2 siRNA and AllStars Negative Control siRNA (SI04948811; 1027280, respectievely; QIAGEN) were transfected using the HiPerFect Transfection Reagent (301704; QIAGEN) according to the manufacturer's instructions. Cells were subjected for further analysis 72h post-transfection.

### Colony formation assay

MDCK naïve cells and stable clones were plated onto 10-cm plates (at three different densities of 1×10^3^, 1.5×10^3^ and 2×10^3^ cells/plate); SKBR-GFP and SKBR-NCL cells were pre-treated with GroA in the indicated concentration for 5 days, and then transferred to 10-cm plates (at three different dilutions of 1:10, 1:20 and 1:40). For all experiments, 7-11 days later, the cells were fixed with 0.1% acetic acid in PBS, and stained with 0.4% crystal violet in 0.1% acetic acid. Total colonies area was calculated using the ImageJ program.

### Anchorage-independent growth assay

2% noble agar was melted and mixed with medium (×2,20% FBS), and the mixture (50 μl) was placed in 96-well plates to provide the base agar (at a final concentration of 1%). The cells were suspended in medium (×2, 20% FBS, mixed with 0.6% noble agar) and 50 μl were plated on the base agar. 100 μl of medium (×1, 10% FBS) were added to the wells. The plates were incubated for 10-14 days at 37°C. Colonies were then stained with 25 μl 3-(4,5- dimethylthiazol-2-yl)-2,5-diphenyltetrazolium bromide (5 mg/ml) and photomicrographed. Number of colonies per well (>0.01 mm^2^) was determined using the ImagePro software.

### Methylene blue viability assay

Naïve and transfected cells were plated in 96-well plates. When relevant, cells were treated as indicated and grown for 4 days for GFP-TM-NLS expression experiments and 3-7 days for GroA treatment experiments. Cell number determination was performed as described previously [39]. IC-10, 20 and 50 values were calculated using a non-linear regression model (logarithmic inhibitor vs. normalized response-variable slope) with the GraphPad Prism 6 software.

### Lysate preparation and immunoprecipitation

After the indicated treatment, cells were lysed in solubilization buffer. Lysates were cleared by centrifugation and a boiling gel sample buffer was added. For immunoprecipitation assays, antibodies were coupled to anti-IgG agarose beads; for Flag-nucleolin precipitation, mouse anti-Flag M2 beads were used (A2220; Sigma-Aldrich). The beads were then incubated with cell lysates. The immunoprecipitates were washed and the proteins were eluted by boiling in gel sample buffer or by addition of Flag peptide (F3290; Sigma-Aldrich), according to the manufacturer's instructions, when relevant. For all immunoblotting, proteins were resolved by SDS-polyacrylamide gel electrophoresis through 10%–12.5% polyacrylamide gels, and were electrophoretically transferred to nitrocellulose membranes. Membranes were blocked in TBST buffer containing 6% milk, and blotted with primary antibodies. Secondary antibody linked to horseradish peroxidase was then added. Immunoreactive bands were detected with the enhanced chemiluminescence reagent.

### Proximity ligation assay (PLA)

For proximity ligation assay (PLA) cells were plated in 16-well Nunc Lab-Tek glass Chamber Slide System (178599; Thermo Scientific) and grown for 48-72h with or without siRNA treatment, as indicated. Following fixation, cells were incubated with rabbit anti-ErbB2 and mouse anti-nucleolin antibodies. PLA was performed using the Duolink *In-Situ* PLA probes: anti-rabbit MINUS and anti-mouse PLUS and the Duolink *In-Situ* Detection Reagents Red kit (DUO92005; DUO92001; DUO92008, respectively; Sigma-Aldrich), according to the manufacturer's instructions. Nuclei were stained using the Duolink *In-Situ* Mounting Medium with DAPI (DUO82040; Sigma-Aldrich). Slides were visualized 24h post-staining and images were obtained using an Olympus motorized inverted research microscope Model IX81 (60× magnification). Signal intensity was determined using ImageJ software.

### DNA constructs

Generation of expression vectors for pEGFP-nucleolin (NCL) and pEGFP-nucleolin variants and GFP-TM-NLS were previously described [8, 22]. ErbB2 ΔCyt-NLS (1-691 a.a.) is a deletion mutant, containing only the extracellular, transmembrane and the NLS domains of ErbB2. The fragment was amplified using PCR, digested with HindIII and KpnI and cloned into a pcDNA3 vector. The primers used to generate this mutant were: 5′-GCC GCT CGA GGA TGA GGA TCC CAA AG-3′ and 5′-GCG-GTA CCT CAC AGC TCC GTT TC-3′. ErbB2-ΔNLS (1-1255 a.a., excluding a.a. 676-690) is the full length receptor, with the exception of the NLS. In order to specifically remove the NLS domain, the part of the gene upstream of the NLS and the part of the gene downstream of the NLS were amplified separately. The upstream part was digested using HindIII and XhoI and cloned into a pcDNA3 vector. The downstream part was digested using XhoI and XbaI and cloned into a pGEM T-easy vector and later into the pcDNA3 vector containing the upstream part. The primers used to generate this mutant were 5′-AGC AAG CTT CGC CAC CAT GGA GCT GGC G-3′ and 5′-GCC GCT CGA GGA TGA GGA TCC CAA AG-3′ for the region upstream of the NLS, and 5′-GAG CCT CGA GCA GGA AAC GGA GCT G-3′ and 5′-GCT CTA GAT CAC ACT GGC ACG TCC AGA CCC AG-3′ for the region downstream of the NLS.

### Statistical and bioinformatical analysis

All experiments were performed at least three times. Results are presented as means ± SD/SE. Differences between means were assessed by the 1-tailed Student's t-test, ANCOVA, one-way ANOVA or two-way ANOVA. Significance was assigned at *p*<0.05.

The bioinformatical data presented are based upon data generated by The Cancer Genome Atlas (TCGA) Research Network: http://cancergenome.nih.gov/. Bioinformatical analyses were performed using MedCalc for Windows, version 12.5 (MedCalc Software, Ostend, Belgium).

## Abbreviations

AML: acute myeloid leukemia
Co-IP: co-immunoprecipitation
DMEM: Dulbecco's modified Eagle medium
ECM: extra-cellular matrix
EGF: Epidermal growth factor
GAR: glycine-arginine rich
IC: inhibitory concentration
MAPK: mitogen-activated protein kinase
NCL: nucleolin
NLS: nuclear localization signal
PBS: phosphate buffered saline
PI3K: phosphoinositide 3-kinase
PLA: proximity ligation assay
RBD: RNA-binding domain
RTK: receptor tyrosine kinase
SDS-PAGE: sodium dodecyl sulfate polyacrylamide gel electrophoresis
TCGA: The Cancer Genome Atlas
